# Cardiac Conduction System: Delineation of Anatomic Landmarks With Multidetector CT

**Published:** 2009-11-01

**Authors:** Farhood Saremi, Maria Torrone, Nooshin Yashar

**Affiliations:** Department of Radiological Sciences of University of California, Irvine

**Keywords:** Cardiac Conduction System, Delineation of Anatomic Landmarks, Multidetector CT

## Abstract

Major components of the cardiac conduction system including the sinoatrial node (SAN), atrioventricular node (AVN), the His Bundle, and the right and left bundle branches are too small to be directly visualized by multidetector CT (MDCT) given the limited spatial resolution of current scanners. However, the related anatomic landmarks and variants of this system a well as the areas with special interest to electrophysiologists can be reliably demonstrated by MDCT. Some of these structures and landmarks include the right SAN artery, right atrial cavotricuspid isthmus, Koch triangle, AVN artery, interatrial muscle bundles, and pulmonary veins. In addition, MDCT has an imperative role in demarcating potential arrhythmogenic structures. The aim of this review will be to assess the extent at which MDCT can outline the described anatomic landmarks and therefore provide crucial information used in clinical practice.

## Introduction

Anatomic and electrophysiological studies have provided strong background on the cardiac conduction system and its electrical connection structures. The role of this system is to ensure rhythmic myocardial stimulation, leading to physiological contraction of the heart. Multidetector CT (MDCT) is emerging as a successful tool for noninvasive, high resolution imaging of cardiac anatomy. In particular, MDCT has an imperative role in outlining and anatomically delineating the cardiac sites related to the conduction system. Since anatomic variation of the cardiac conduction system landmarks and associated structures is common, it is crucial to learn more about these normal  variants, especially prior to interventional procedures.

## Cardiac Conduction System

The cardiac conduction system is composed of the sinoatrial node (SAN), the atrioventicular node (AVN), the HIS bundle, the right and left bundle branches, the fascicles and the Purkinje fibers [1-4]. The conduction system  consists of specialized myocytes. Its atrial components, the SAN (subepicardial) and the AVN (subendocardial), are in contact with the atrial myocardium [1,2]. While no morphologically distinct conduction pathway between the SAN and AVN is demonstrable, functional pathways due to geometric arrangement of working muscle fibers could be responsible for the conduction between the two structures along certain preferential routes [2,3]. The His bundle goes through  the right fibrous trigone (central fibrous body) and runs at the junction of the membranous and muscular septum before it divides into the right and left ventricular bundle branches [4]. The right bundle branch is a cord like structure with a 1 mm diameter, which proceeds along the septal and moderator bands to reach the anterior papillary muscle. In contrast,  the  left bundle branch forms a broad sheet of conduction fibers that splits along the left side of interventricular septum into three indistinct fascicles [5].

## Related Anatomic Structures

### Right atrium

**Figure 1:**
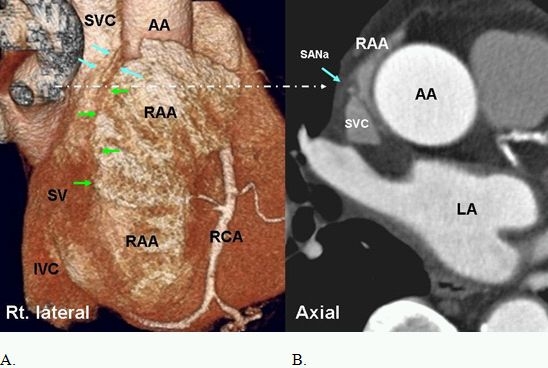
A. Right lateral volume rendered image of the heart shows the terminal segment of SA node artery (SANa) (blue arrows) in the sulcus terminalis (green arrow). Large right atrial appendage (RAA) with irregular surface due to prominent pectinate muscles is seen. B. Axial view at the approximate level of the SA node (long arrow) demonstrates central position of the SANa (blue arrow) within the crista terminalis at superior cavoatrial junction. The SA node is arranged around the SANa. AA=ascending aorta, LA=left atrium, RCA=right coronary artery, IVC=inferior vena cava, SV=sinus venarum

The right atrium (RA) has 3 components: an appendage, a venous component (sinus venosus) and a vestibule [1].  The crista terminalis, a prominent muscular ridge, separates the appendage from the sinus venosus [6,7]. The vestibule is a smooth muscular rim surrounding the tricuspid orifice. The terminal groove, or sulcus terminalis, is a fat filled groove on the epicardial side that corresponds internally to the crista terminalis. The SAN and the terminal segment of the SAN artery are located in this groove, close to the superior cavoatrial junction [6] (Figure 1). Sinus Venosus is located mainly in the posterolateral wall of the RA between the orifices of the superior vena cava (SVC) and inferior vena cava (IVC).

### Interatrial septum

**Figure 2:**
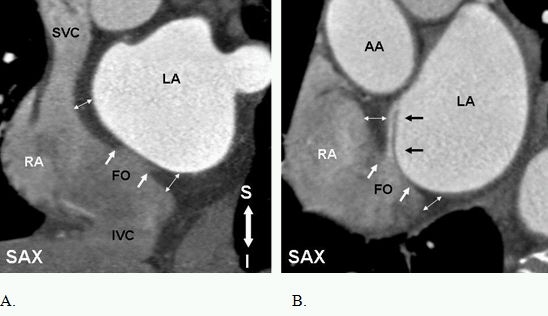
Anatomy of the interatrial septum and patent foramen ovale (PFO). Short axis (SAX) images perpendicular to the interatrial septum show the fossa ovale (FO) in (A) and a well structured PFO in (B). The septum primum (black arrows in B) is fused to the inferior rim of fossa ovale (FO) and extends superiorly as a flap. The superior and the inferior rims (white arrows) of the FO are formed by infolding of the right atrial wall [interatrial groove (IAG) or the septum secundum]. IAG (double headed arrows) contains extracardiac fat. The infolding of the right atrial wall overlaps the flap of septum primum, forming a narrow tunnel through which a probe can be passed (51). AA= ascending aorta, LA=left atrium, RA=right atrium, S=superior, I=inferior. Reproduced  from: Saremi F, Tafti M. The role of computed tomography and magnetic resonance imaging in ablation procedures for treatment of atrial fibrillation. Semin Ultrasound CT MR. 2009;30:125-56, Copyright (2009), with  permission from Elsevier.

**Figure 3:**
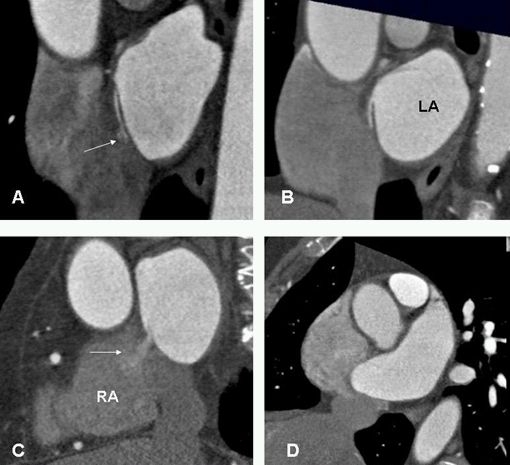
Short axis images at the level of fossa ovalis demonstrate different anatomic variants. A. PFO with small left to right shunt (arrow). B. flap valve closed at the point of entry into the right atrium. This variant is seen in 15% of individuals. C. Incompetent valves with free flow of contrast from left to right (arrow). Note, the flap valve is too short causing valve incompetency. D. Atrial septal aneurysm. LA=left atrium, RA=right atrium.

The true atrial septum is made up of the flap valve of the foramen ovale (septum primum) and part of its anteroinferior margin (Figure 2). The superior rim of the fossa or the septumsecundumis the infolded wall between the SVC and the right pulmonary veins (PV). This is referred to as the interatrial groove and is not a true septum. Incomplete fusion of the flap of the foramen ovale against the atrial septum results in a probe patent defect, or patent foramen ovale (PFO). The PFO is usually less than 5 mm in diameter [8]. Pre-procedural anatomic knowledge of the atrial septum can minimize complications of transseptal approaches [9]. A PFO is often associated with atrial septal aneurysm and Chiari network [9,10]. Anantomic variants of this complex anatomy can easily be assessed with MDCT (Figure 3).

### The septal components of the AV junction

Conducts the cardiac impulse from the atria to the ventricles [8]. The central fibrous body (apex of Koch triangle) lies superior and anterior to the muscular AV septum. The central fibrous body, made up of the the right fibrous trigone and the membranous septum, fuses together the aortic, mitral and tricuspid valves.

### Left atrium

The left atrium (LA) like the right atrium consists of an appendage, a venous component, and a vestibule [3,11]. The left atrial appendage is derived from primitive atrium and has a rough, trabeculated surface. It is a potential site for thrombus deposition  due to itsnarrow neck with the LA. The venous component  has pulmonary vein orifices at each corner and  is located posteriorly. The vestibular component surrounds the mitral orifice. The greater portion of the LA, which includes the venous component, the vestibule and the septal component is smooth walled.

### SAN

**Figure 4:**
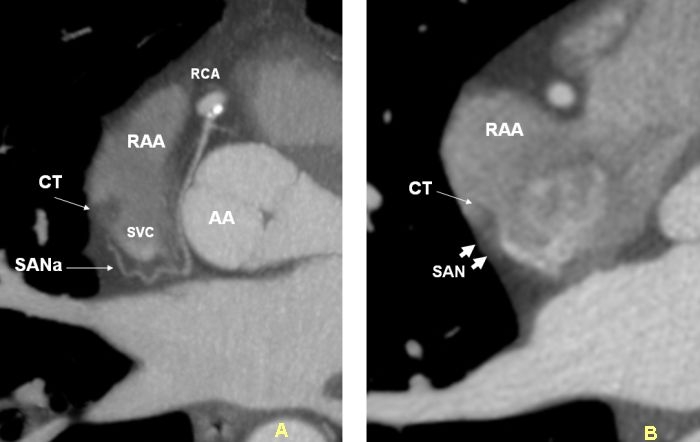
Axial images in two different patients. A. demonstrates the arterial supply of the sinoatrial node artery (SANa) arising from the proximal right coronary artery (RCA). Terminal segment of the SANa passes behind the superior vena cava (SVC) (retrocaval). Retrocaval course occurs in 47% of individuals. B. The terminal portion of the SANa is not seen. However enhancing SA node (SAN) (small arrows) can be seen in epicardial aspect of the crista terminalis (CT). CT is partially infiltrated by fat. AA=ascending aorta.

The SAN is a subepicardial, spindle shaped structure at the superior cavoatrial junction that extends along the crista terminalis toward the IVC [7,12-14]. It gradually penetrates musculature of the crest to rest in the subendocardium. The SAN surrounds the SAN artery, which can course centrally (70%) or eccentrically within the node [7] (Figure 1). Histologically, it is composed of cells slightly smaller than normal working cells [13,15]. The SAN varies in position and length along the crista terminalis. Mean length of the SAN is reported as 20 ± 3 mm  [15]. With age, the amount of connective tissue increases with respect to the area occupied by the nodal cells [16]. The approximate location of the SAN can be localized in axial CT images by locating the SA node artery along the crista terminalis (Figure 4).

### Crista Terminalis

**Figure 5:**
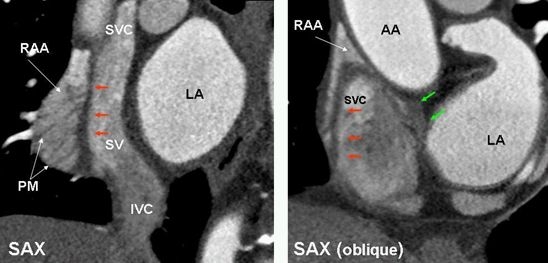
Short axis images in two different patients demonstrate the crista terminalis (red arrows) as a dark band between right atrial appendage (RAA) and sinus venarum (SV) extending from the superior vena cava (SVC) to the inferior vena cava (IVC). Superiorly, the CT arches anterior to the orifice of the SVC and extends to the area of the anterior interatrial groove and merges with the interatrial bundle, commonly known as Bachmann's bundle (green arrows). Prominent pectinate muscles (PM) are seen.  LA=left atrium, AA=ascending aorta.

The crista terminalis is a fibromuscular ridge formed by the junction of the sinus venosus and the primitive RA [1,4]. Superiorly it arches anterior to the orifice of the SVC, extends to the area of the interatrial groove, and merges with the interatrial bundle, commonly known as the Bachman bundle (Figure 5). The inferior border of the crista terminalis near the IVC orifice is indistinct and merges with small trabeculations of the inferior portion of the cavotricuspid isthmus [7]. The crista terminalis gives rise to a series of relatively thick bundles known as the anterior pectinate muscles, which fan out anteriorly. The septum spurious is the most prominent anterior pectinate muscle. It is present in 80% of hearts and can measure up to 4.5 mm, and should not be mistaken for interatrial disease [7]. MDCT can be used to measure the thickness of the crest and demonstrate the approximate location of SAN artery within the nodal tissue. Since the crista terminalis is linked to several forms of atrial tachyarrhyhmias a relationship between the thickness of the crista terminalis and the development of atrial flutter  could exist [17].

### AVN

The AVN lies in the Koch Triangle. It  includes a compact portion and an area of transitional cells [18]. The AVN continues distally with the penetrating His bundle. The His bundle is surrounded by the connective tissue of the central fibrous body and is therefore a conducting tract that takes information to the ventricles [18].

### Koch triangle

The Koch triangle  rests in the RA,  anterior to the orifice of the coronary sinus. The apex of the Koch Triangle is the central fibrous body of the heart, where the His bundle also penetrates. It is bordered posteriorly by a fibrous extension from the eustachain valvae called the tendon of Todaro [19]. The anterior border is demarcated by the attachment of the septal leaflet of the tricuspid valve. The midportion of the triangle contains the compact AV node (fast pathway) and the base contains the slow pathway. The base of the triangle is bordered by the coronary sinus ostium and anteriorly by the septal isthmus.

## Vascular Supply

### SAN artery

The SAN artery  comes off of either the proximal right coronary artery (60-70%) or the proximal circumflex artery  [12,13,20] (Figure 4). In less than 1% of human hearts, the SAN artery may originate directly from the right coronary sinus, descending aorta, or distal right coronary artery. Knowledge of these anatomic variants can be important prior to surgery [21,22]. Information regarding the termination of the SAN artery may be imperative when planning a superior transseptal approach in mitral valve surgery [23,24]. The SAN artery crosses the superior posterior border of the interatrial septum in 54% of hearts. MDCT data has shown that the terminal SAN artery travels closer to the superior aspect of the interatrial septum in selected groups when the artery is moving behind the cava (47%) [20]. Such anatomy predisposes the SAN artery to injury during a superior transseptal approach to the mitral valve.

**Figure 6:**
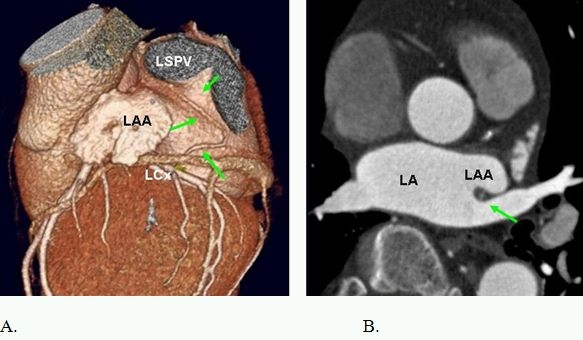
Anatomic course of the S-shaped SAN artery. A. Arising from the proximal left circumflex artery (LCx), it turns posteriorly and moves in the groove between the left atrial appendage (LAA) and the left superior pulmonary vein (LSPV) orifices (short arrows). 30% of SAN arteries arising from the LCX are S-shaped in their anatomic course.

Another  significant variant of the SAN artery is the existence of a left S-shaped SAN artery arising from the proximal LCx, seen in 8% of the cadaveric hearts studies [25] and in 14% of the coronary CT studies [26] (Figure 6). This artery is larger than the normal SAN artery, and supplies almost the whole left atrium, a large part of the interatrial septum and right atrium, a part of the sinus and the atrioventricular nodal areas. The superb resolution of MDCT provides definitive localization of this artery, where it passes in the sulcus between the left superior pulmonary vein and the left atrial appendage [26]. In this location, the artery becomes susceptible to injury during catheter or surgical ablation procedures on the left atrium.

### AVN artery

**Figure 7:**
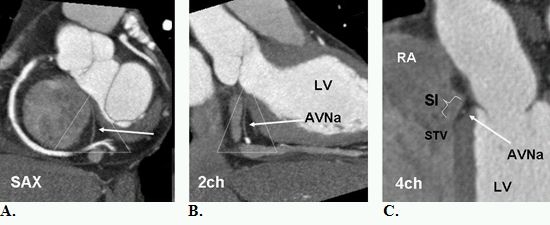
A. B., and C. Inferior pyramidal space (dotted triangle) is the anatomic location of atrioventricular node artery (AVNa). The AVNa originates from the distal right coronary artery (RCA) and penetrates into the base of the posterior interatrial septum. The AVNa supplies the AV node at the apex of the pyramid, in close proximity to the muscular atrioventricular septum. This area is not a true septum but extracardiac fat sandwiched between the right atrium (RA) and the left atrium (LV) (arrow in c). The septal isthmus (SI) is shown in (C) extending between the septal tricuspid valve (STV) and coronary sinus ostium. The AVNa is in potential danger when ablation procedure of the septal isthmus is performed. SAX=short axis view, 2ch= two chamber view, 4ch=four chamber view, LV= left ventricle. SI=septal isthmus (bracket).

The AVN artery derives from the apex of the U turn of the distal RCA and invades the base of the posterior interatrial septum (inferior pyramidal space) at the level of the crux of the heart in 80-87% of patients [20,27-29] (Figure 7). In the remaining population, the artery originates from the terminal portion of LCx artery (8 - 13%) or uncommonly from both RCA and LCx (2 - 10%). The artery provides branches to the posterior interventricular septum, interatrial septum, AVN, and penetrating bundle of His [18].  In some patients, at the level of the Koch triangle, the AVN artery courses just beneath the endocardium near the ostium of the coronary sinus and the septal isthmus (Figure 7). This may explain the higher risk of AVN artery coagulation during radiofrequency ablation in the slow pathway region, while complete AV block is commonly a direct result of tissue injury to the AV node [30].

### Alternative sources of arterial supply to the atrioventricular conducting pathway

These include the first septal perforating artery, the descending septal artery, and anterior atrial branches which take into account the Kugel anastomotic artery [18,20,31,32]. The Kugel anastomotic artery was first described by MA Kugel [33] as a large atrial artery (arteria anastomotica auricularis magna). This artery is a rare, but an important collateral between the proximal LCx or RCA (3%) and for whichever artery that supplies the crux of the heart (distal RCA or distal LCx). It passes anterior to the mitral valve ring, coursing in the lower interatrial septum and may anastomose with the AVN artery. The right Kugel anastomotic artery [32] may be a continuum of either the right superior septal vessel, a branch of SAN artery or conus branch [34,35]. The  first septal perforating artery is a branch of the left anterior descending coronary artery which supplies the basal septum with divisions to the conduction system including His bundle and proximal bundle branches [36]. It is not a primary arterial source to AV node, but its terminal branches can connect with right superior septal artery. It is known that complete heart block is common after alcohol septal ablation for the treatment of hypertrophic obstructive cardiomyopathy  [37].

## Role of interatrial myocardial connections

### Anterosuperior interatrial connection (Bachmann's bundle)

**Figure 8:**
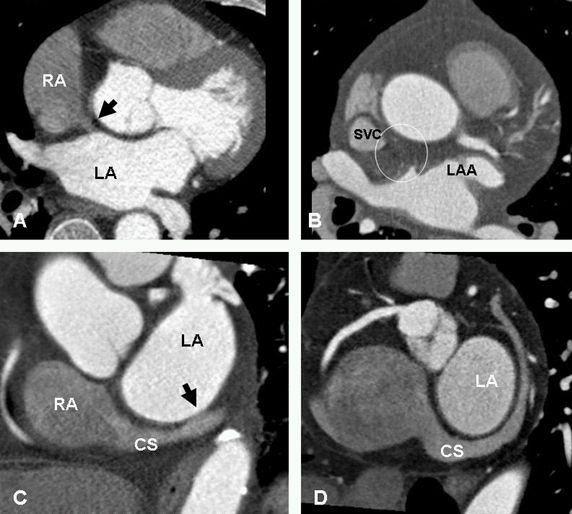
Variants of Bachman bundle (BB) and CS-LA muscle continuity. A. BB is shown (black arrow) connecting the two atria anteriorly up to the junction of the superior vena cava (SVC) in the right atrium (RA) and extending to the left atrial appendage (LAA) in the left atrium. B. Shows complete replacement of BB by fat (within the circle). C. Proximal CS-LA attachment (black arrow) near the junction with the great cardiac vein. D. No CS-LA attachment. Fat fills the space between the CS and left atrial wall. CS=coronary sinus, LA=left atrium.

Bachmann's bundle (BB), the preferential interatrial electrical connection structure, ensures rapid interatrial conduction, and therefore leads to physiological biatrial contraction [38]. It is a subepicardial flat band of muscle fibers at the anterosuperior margin of the interatrial groove (Figure 8). The SAN artery and its branches are the principal vascular supply of BB [39]. Changes in the musculature of BB could block or prolong interatrial conduction resulting in abnormal atrial excitability, atrial dysfunction, AF, and other arrhythmias [40]. Although BB and its vascular supply can easily be detected by 64-MDCT, BB is less visible in patients with severe coronary artery disease, atrial fibrillation, and interatrial conduction block [40]. In the absence of BB, the area is replaced by fat, which may suggest an association between these conditions and the diseased BB fibers.

### Posteroinferior interatrial connection and the Coronary Sinus

In addition to the anterosuperior interatrial muscle bridge of BB, there are other muscular bridges of variable numbers and sizes that provide interatrial connections [41]. The coronary sinus is approximately 30-45 mm long and 10-12 mm in diameter, with highly variable morphologic features [42,43]. The beginning of the coronary sinus is marked by either an outer constriction, an opening of the oblique vein of Marshall, or internally by the Vieussens valve. The CS is surrounded by a striated myocardial sleeve outside its tunica adventitia, which continues into the right atrium [41,44]. This myocardial extension into the CS is electrically continuous at one or more points to the right and left atria. In many instances, MDCT can show the continuity of this myocardial coat with musculature of the left atrium (Figure 8).

The coronary sinus is used as a conduit for catheter treatment of arrhythmias [43-45]. Anatomic variants of the CS exist, including diverticulum, stenosis, ectasia, unroofed sinus, or atresia [6]. The majority of coronary sinus diverticula are located along its inferior aspect, usually at its junction with the middle cardiac vein [45]. A CS diverticulum may form the anatomic basis of posteroseptal or left posterior accessory pathways [45]. A coronary sinus diverticulum differs from a subthebesian pouch, which is a recess of the right atrial CTI extending below the orifice of the coronary sinus. Proximal CS in patients with AV junctional reentry tachycardia is shown to be larger than in healthy patients and resembles a wind sock [46].

## Anatomic landmarks related to arrhythmias

### Cavotricuspid Isthmus

**Figure 9:**
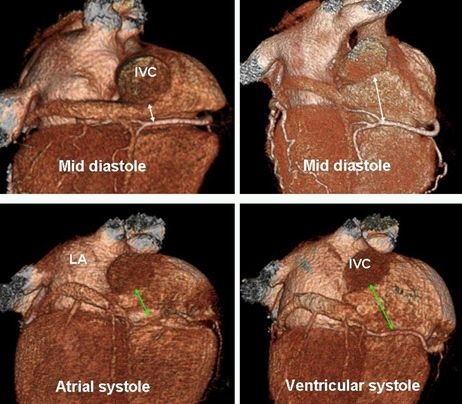
3D posterior views of the heart. The length of the CTI varies in different individuals (upper panel, different patients) and different cardiac phases (lower panel, same patient). Knowledge of these anatomic variants prior to catheter ablation for atrial flutter will save time and increases the success rate.

The right atrial cavotricuspid isthmus is  the area between the IVC and tricuspid valve. This site is the target of catheter ablation techniques that have become the treatment of choice for isthmus dependent atrial flutter. The size of this region varies differently among individuals and  across the phases of the cardiac cycle (Figure 9). Many anatomic obstacles such as an enlarged Eustachian ridge, aneurismal pouches, or even a concave deformation of the entire isthmus, can make ablation difficult.

### Subthebesian pouch

**Figure 10:**
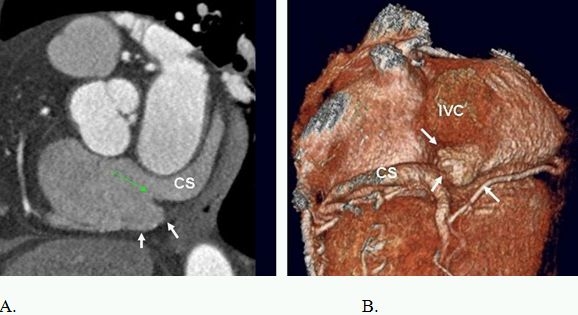
Short axis (A) and volume rendered (B) images show a large subthebesian pouch (white arrows) extending beneath the coronary sinus (CS) orifice and the Thebesian valve (green arrow).

The atrial wall inferior to the orifice of the coronary sinus is usually pouchlike and  described as the sinus of Keith, or subeustachian sinus [2,7]. It is anterior to the orifice of the IVC and is subthebesian rather than subeustachian (Figure 10). It has a special arrangement of muscle fibers which can be the substrate for the reentrant circuit during atrial flutter. The depth of the subthebesian pouch can be a cause of procedural difficulty.

### Pulmonary Veins

**Figure 11:**
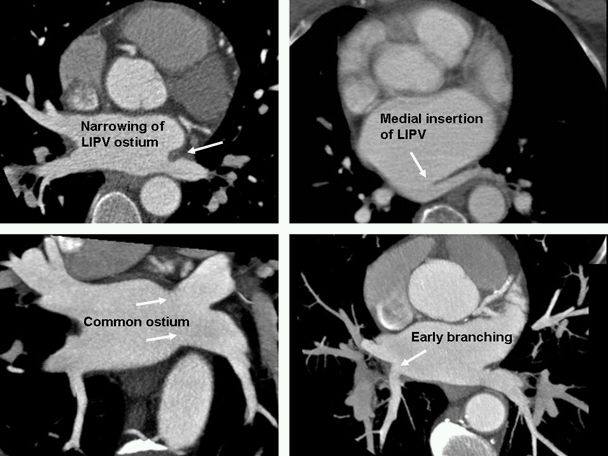
Anatomic variants of the pulmonary vein insertion. It is not uncommon to see mild narrowing of the left inferior pulmonary vein (LIPV) at its confluence with the left atrium. This is most likely secondary to the compressive effect of the pulsating aorta and should not be mistaken for stenosis after radiofrequency ablations. Medial insertion of the LIPV is relatively rare and may cause difficulty for circumferential pulmonary vein isolation. Common ostium is common and can happen on either left or right side. Early branching is also common and usually is seen with right upper lobe pulmonary vein entering near the confluence of right superior pulmonary vein with the left atrium.

It is well established that myocardial sleeves of the PVs in particular the superior veins are crucial sources of triggers, which initiate atrial fibrillation (AF) [47-49]. Imaging studies have demonstrated that the anatomy of the LA and PVs is commonly variable [50-53] (Figure 11). The PV ostia are ellipsoid with a longer supero-inferior dimension. Veins are larger in AF versus non-AF patients, men versus women, and persistent versus paroxysmal patterns. The PV trunk is defined as the distance from the ostium to the first order branch. The superior pulmonary vein ostia are larger (19-20 mm) than the inferior pulmonary vein ostia (16-17 mm) [50-53]. It is important to report the ostial diameters of each vein and the length to the first order branch because these measurements influence the selection of circular catheter size.

Conjoined (common) PV is very common (> 25%) and more frequently seen on the left than the right. In addition, the supernumerary veins are also  visualized. The most common is a separate right middle pulmonary vein (25%), which drains the middle lobe of the lung [54]. One or two separate middle lobe vein ostia can be seen in 26% of patients. The ectopic focus originating from the right middle PV could initiate AF, which is cured by catheter ablation of right middle PV. In some patients, a supernumerary PV exists, which shows an aberrant insertion with a perpendicular position in relation to the LA posterior wall. Supernumerary branch usually drains the upper lobe of the right lung and characteristically passes behind the bronchus intermedius.

### Septal Isthmus

The septal isthmus is part of the right atrial vestibule located between the edge of the coronary sinus ostium and the attachment of the septal tricuspid valve (Figure 7). This is often the target for ablation of the slow pathway in AV node reentrant tachycardia [17]. It is also the target for ablation of isthmus dependent atrial flutter.

## Other potential arrhythmogenic structures

### Ligament of Marshall and Left SVC

**Figure 12:**
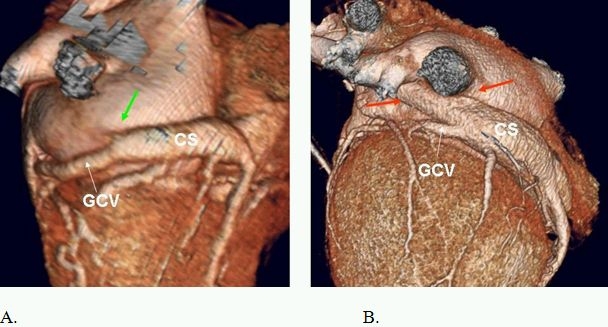
A. Oblique vein of Marshall (green arrow) versus B. persistent left superior vena cava (red arrow). CS=coronary sinus, GCV=great cardiac vein.

In most hearts (70%) the oblique vein or ligament of Marshall (developmental remnant of the embryonic left SVC) is < 3 mm from the endocardium of the left lateral ridge of the LA and contains  muscular connections to the left PVs [55,56]. The remnant of the oblique vein can be detected on coronary CT studies (Figure 12). It remains patent as an isolated malformation, the persistent left SVC draining into the CS, in 0.3% of the normal population and can be the source of atrial fibrillation [56,57].

### Lipomatous hypertrophy of the interatrial septum

MDCT can also be used to help diagnose lipomatous hypertrophy of the interatrial septum, which is characterized by accumulation and deposition of fat in the interatrial septum. The condition commonly occurs in older, obese women.  While in  most case it is  asymptomatic,  it is important to note that it can cause atrial arrhythmias or obstructive flow symptoms [58,59].

### Cardiac Autonomic Nervous System (Ganglionic Plexi)

**Figure 13:**
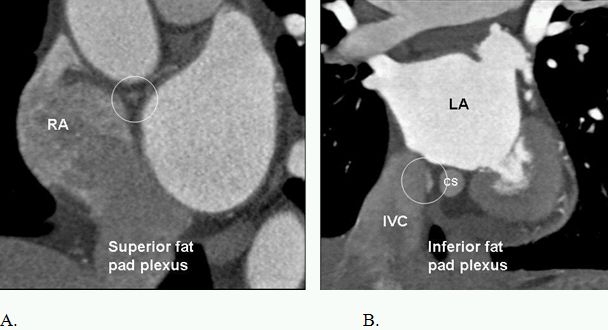
Parasympathetic ganglionic plexi of the heart (within the circles). It is difficult to image vagus plexi of the heart. However, those can indirectly be localized by enhancement of their rich vascular supply. Multiple ganglia exist. The largest collection are located in the fad pad of superior (A) and inferior cavo-atrial junctions (B). Surgical or catheter ablation of these structures have been used for successful ablation of atrial fibrillation. CS=coronary sinus, RA=right atrium, LA=left atrium, IVC=inferior vena cava.

Cardiac ganglia are generally located in the epicardial layer and are surrounded by adipose tissue. The largest populations can be detected with CT (Figure 13) and are concentrated along the interatrial groove near the SA (SVC-right pulmonary vein fat pad) and AV nodes (IVC-LA fat pad) [60-62]. Smaller collections are located on the superior and anterior left atrial surfaces, the atrial appendage-atrial junctions, the base of the great vessels, and the base of the ventricles. Vagal stimulation shortens the atrial effective refractory period that facilitates the initiation and maintenance of AF. By  adding the LA ganglion plexus to other ablation targets, may improve ablation success in patients undergoing circumferential PV ablation for paroxysmal AF [63].

### Assessment of Scar as trigger for reentry tachycardia

Ablation treatment of ventricular tachycardia secondary to myocardial scars due to old myocardial infarctions or cardiomyopathic processes can be challenging becausethe critical parts of the circuit may be difficult to localize  [64]. Post contrast MRI is probably more sensitive than CT in showing the area of a myocardial scar in patients with VT [65]. However, CT does have  the potential to show myocardial scars, especially scarsin the left ventricle.
